# Targeting LRH-1/NR5A2 to treat type 1 diabetes mellitus

**DOI:** 10.15698/cst2018.06.140

**Published:** 2018-05-22

**Authors:** Nadia Cobo-Vuilleumier, Petra I. Lorenzo, Benoit R. Gauthier

**Affiliations:** 1 Andalusian Center for Molecular Biology and Regenerative Medicine-CABIMER, Junta de Andalucia-University of Pablo de Olavide-University of Seville-CSIC, Seville, 41092, Spain.

**Keywords:** LRH-1, NR5A2, immune tolerance, islet regeneration, trans-differentiation, therapy

## Abstract

Type 1 diabetes mellitus (T1DM) is defined as an autoimmune disease that targets the selective destruction of islet insulin-producing beta cells by infiltrating immune cells (insulitis). As a result, the organism is no longer able to produce insulin and develops hyperglycaemia and, if untreated, death. Despite advances in medical device technology and insulin analogues as well as strives in generating *in vitro* insulin-producing cells, there is still no robust therapy to substitute and protect beta cells that are lost in T1DM. Clinical trials aimed at blocking the immune-mediated beta cell destruction have had moderate success leaving a gap in our understanding of disease aetiology. Such breach in knowledge may stem from the oversight that inhibiting the immune attack likely impairs beta cell regeneration and emphasizes a fundamental paradigm in the approach to treat the disease: A non-mutually exclusive strategy in which the uncontrolled self-directed inflammatory immune response (and not the global immune system) as well as beta cell regeneration are exquisitely fine tuned in order to successfully regain immunological tolerance and restoration of a functional beta cell mass. As such, defining factors that can guide a pro-inflammatory immune cell destructive environment towards an anti-inflammatory environment facilitating beta cell survival and stimulate regeneration would define an unprecedented class of immune-regenerative therapeutic agents for T1DM. In our recent study we identify the liver receptor homolog 1 (LRH-1, also known as NR5A2) as a ‘druggable’ target that fulfills these criteria restoring glycemic control in various mouse models of T1DM as well as improving human islet survival and function both *in vitro* and *in vivo *(Nat Comms, 9:1488).

The liver receptor homolog-1 (LRH-1) is a member of the NR5A family of nuclear receptors that plays a pivotal role in early embryonic development while later on it specifies the endodermal lineage. In adults, LRH-1 regulates expression of genes involved in cholesterol, bile acid and glucose metabolism as well steroid biosynthesis. In addition, LRH-1 conveys a cell protective role by attenuating the hepatic acute phase response that is triggered upon increases in pro-inflammatory cytokines as well as to protect against endoplasmic reticulum stress. LRH-1 was also shown to regulate intestinal immune homeostasis through local production of glucocorticoids (GCs) and to mediate the anti-inflammatory and antifungal response of IL-13 activated macrophages. We previously, demonstrated that LRH-1 is expressed in human and rodent pancreatic islets. Viral-mediated over expression of LRH-1 in human islets conferred protection against stress-induced apoptosis correlating with increased expression levels for genes involved in glucocorticoid biosynthesis. Given the role of LRH-1 function in attenuating inflammatory processes as well as in islet survival, we reasoned that attempts to regulate its activity could be of therapeutic value for the treatment of autoimmune diseases such as T1DM.

Towards this goal, we explored the therapeutic value of a small chemical LRH-1 agonist codenamed BL001 in three different mouse models of T1DM: the RIP-B7.1 mouse in which experimental autoimmune diabetes is induced by immunization (IMM) with the preproinsulin cDNA, the spontaneous and random non obese diabetic (NOD) mouse and the streptozotocin (STZ)-induced diabetes mouse model. We found that BL001 treatment either prophylactically or therapeutically reduced the incidence of diabetes in all mouse models by fostering a crosstalk between islet and immune cells that promoted a local anti-inflammatory environment favourable for beta cell regeneration and survival. In support of this BL001-mediated immune-islet cell dialogue we show in the RIP-B7.1 mouse model that BL001 promoted expansion of regulatory T cells (Tregs) as well as T helper 2 (Th2) cells and primed macrophages towards the immunosuppressive and tissue remodelling M2 phenotype. Accordingly, IL10 and TGFbeta circulating levels were also increased in BL001-treated mice correlating with the presence of a larger number of indoleamine 2,3-dioxygenase (IDO)^+^ dentritic cells, which convey immunosuppressive and tolerogic functions. Silencing of LRH-1 in mouse macrophages blocked the BL001-mediated M2 genetic signature and blunted IL-10 secretion validating the direct and specific role of the BL001/LRH-1 signalling pathway in establishing the macrophage anti-inflammatory phenotype. In parallel, BL001 directly stimulated the secretion of anti-inflammatory GCs from islets that likely results in further propagation of Tregs and M2 polarization correlating with insulitis resorption. Historically, GCs were shown to induce insulin resistance, to be diabetogenic and to hamper islet function *in vivo*. These findings led to their exclusion from immuno-suppressive regimens used subsequent to human islet transplantation. However, our study, along with others, demonstrating that GCs considerably improved glucose-induced insulin secretion of transplanted human islets *in vivo*, is now challenging this dogma.

Our work also unravelled that agonistic activation of LRH-1 stimulates beta cell regeneration, a process that appears to be mediated by alpha-to-beta cell trans-differentiation. In support of this premise, a remarkable number of cells expressing both glucagon and insulin were detected in IMM and BL001-treated mice correlating with an increase in beta cell mass without a significant effect on proliferation. Furthermore, key transcription factors involved in the alpha cell genetic program were repressed in an alpha cell line treated with BL001 substantiating the role of the LRH-1 signalling pathway in cell conversion. Detection of such bi-hormonal cells expressing insulin and glucagon in non-diabetic insulin-resistant patients pinpoint to an important role for alpha-to-beta cell trans-differentiation in human islets with low proliferative capacity in order to cope with higher insulin demands. The prospect that BL001 induces human alpha cell conversion is highly relevant in the context of a T1DM therapy as patients exhibit scarce functional beta cells refractory to proliferation signals while they harbour abundant alpha cells that could be reprogrammed towards insulin-producing cells.

The marked increased in glucagon/insulin expressing cells induced by BL001 in IMM mice as compared to the non-immune-mediated STZ mouse model of beta cell ablation prompt us to propose that Tregs and M2 macrophages contribute to alpha-to-beta cell trans-differentiation. It is well established that Tregs and M2 macrophages are key remodelling players in mouse tissue repair (muscle, bone and vasculature) promoting cell differentiation and expansion. This integrated non-mutually exclusive and mandatory islet-immune dialogue may provide some clues on failures of clinical trial targeting solely the immune system that will likely compromise beta cell replenishment.

Transcriptome profiling of human islets treated with BL001 revealed alteration in the expression of genes involved in beta cell survival. Consistent with this premise BL001-treated human islets were resistant to cytokine-induced apoptosis. Furthermore, BL001 treatment improved glucose-induced insulin secretion of islets isolated from type 2 diabetic donors through increased beta cell survival. More importantly, the integrity of human islets xenotransplanted in immune competent C57BL7/6 mice was preserved under BL001 treatment. Thus, agonistic activation of LRH-1 not only induces immune tolerance but also enhances beta cell survival and regeneration resulting in the reversion of T1DM.

Identifying unique ‘druggable’ targets that ‘re-educate’ an ongoing autoimmune attack towards ‘one-self’ tolerization while promoting regeneration of a functional beta cell mass offer a promising mono therapy for T1DM. To date such target or pharmacological agents intervening at the interface between immunity and islet plasticity remain at large. Our work provide strong evidence that activation of the nuclear receptor LRH-1/NR5a2 using a small chemical agonist, fulfill these requirements and reverts diabetes in several mouse models via an intricate cross talk between islet and immune cells (**Figure 1**). In light of these results, development of more potent and stable second-generation agonists of LRH-1 that can be administered orally (BL001 having a short half-life via this route), rather than daily intraperitoneal injections, may prove to be a feasible therapeutic alternative and/or complement to insulin treatment for T1DM patients.

**Figure 1:**
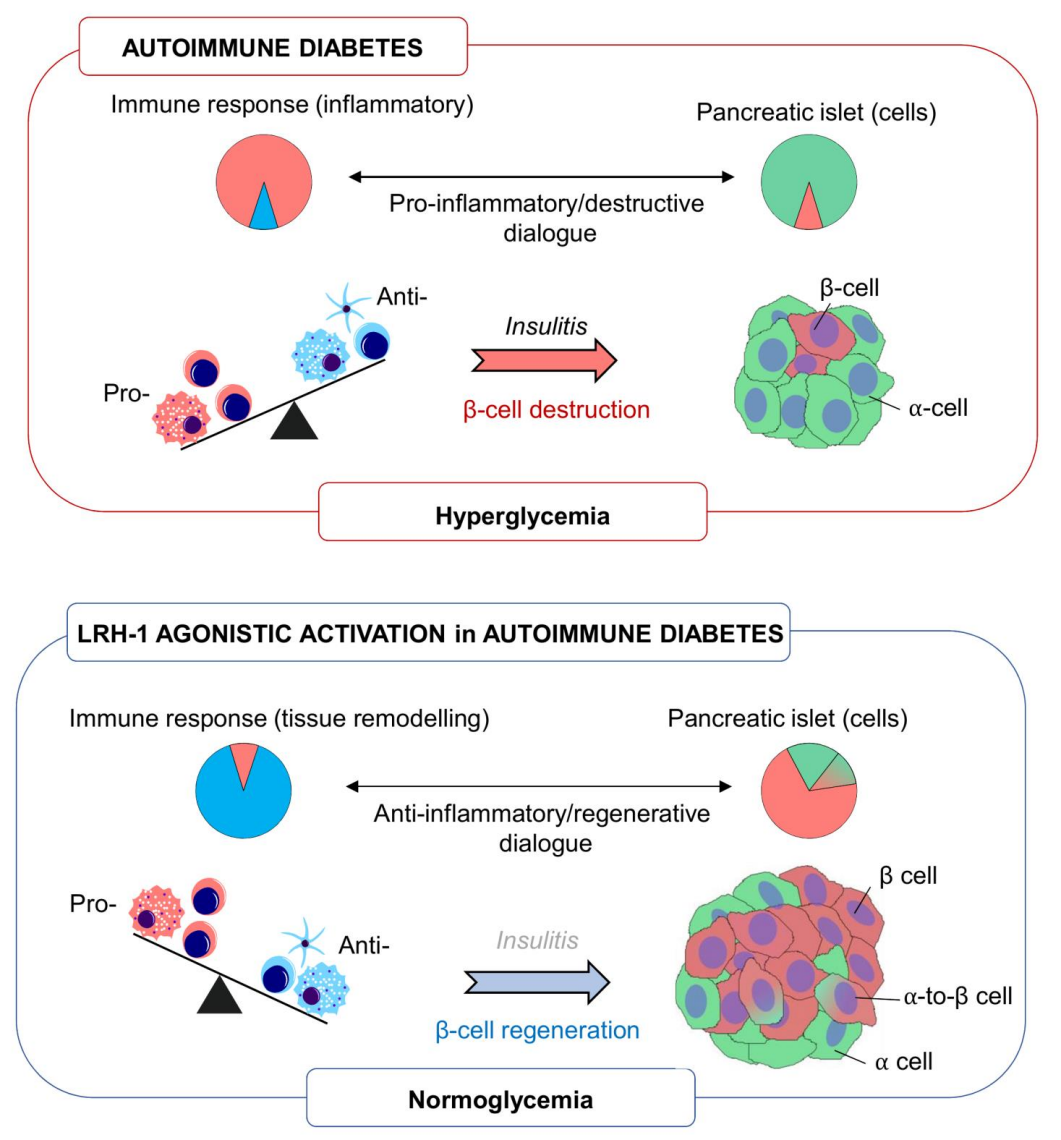
FIGURE 1: Schematic overview of LRH-1 agonistic activation in favouring and anti-inflammatory and pro-islet regenerative environment implicating alpha-to-beta cell transdifferentiation. Pie charts depict the relative percentage of either immune or islet cells under the various conditions.

